# Modelling and simulation of waste tire pyrolysis process for recovery of energy and production of valuable chemicals (BTEX)

**DOI:** 10.1038/s41598-023-33336-3

**Published:** 2023-04-13

**Authors:** Yan Cao, Ali Taghvaie Nakhjiri, Shahin Sarkar

**Affiliations:** 1 School of Computer Science and Engineering, Xi’an Technological University, Xi’an, 710021 China; 2 Department of Petroleum and Chemical Engineering, Science and Research Branch, Islamic Azad University, Tehran, Iran; 3 Department of Applied Science, Technological University of the Shannon, Midlands Midwest, Moylish, Limerick, V94 EC5T Ireland

**Keywords:** Chemical engineering, Energy science and technology

## Abstract

The pyrolysis oil fraction is highly attractive for pyrolysis products. A simulated flowsheet model of a waste tire pyrolysis process is presented in this paper. A kinetic rate-based reaction model and equilibrium separation model are created in the Aspen Plus simulation package. The simulation model is effectively proven against experimental data of literature at temperatures of 400, 450, 500, 600 and 700 °C. Also, the developed model was employed to investigate the impact of temperature on the pyrolysis procedure and demonstrated that there is an optimum temperature for chain fractions. The optimum temperature to have the highest amount of limonene (as a precious chemical product of waste tire pyrolysis process) was found 500 °C. The findings indicated that the pyrolysis process is ecologically benign, although there is still space for development. In addition, a sensitivity analysis was carried out to see how altering the heating fuel in the process would affect the non-condensable gases produced in the process. Reactors and distillation columns in the Aspen Plus^®^ simulation model was developed to assess the technical functioning of the process (e.g., upgrading the waste tires into limonene). Furthermore, this work focuses on the optimization of the operating and structure parameters of the distillation columns in the product separation unit. The PR-BM, as well as NRTL property models, were applied in the simulation model. The calculation of non-conventional components in the model was determined using HCOALGEN and DCOALIGT property models.

## Introduction

Globally, rising waste tire production is posing a significant economic and environmental concern^1^. Natural resource depletion and crude oil depletion, from which synthetic rubbers are made, are also economic issues^2–7^. Eco-friendly issues are mostly associated with the enormous piles of stocked waste tires^3,4,6–11^. Waste tires have been used in processes such as retreating, grinding, incineration, material recovery, energy recovery and pyrolysis^12^. Blending crumb rubber beside asphalt for highway formation, burning for power and/or steam production, and reprocess in the making of plastic and rubber products as a filler are all traditional strategies for reducing waste tire stocks^13^. Nevertheless, these methods are matured as they are charged with economic—high capital and operating costs of amenities—and environmental tasks—toxic compounds emissions. The conventional technique for treating waste tires is to employ tire-derived-fuel (TDF) for energy recovery, with the bulk of TDF being used in cement kilns. This application has some limitations, such as control of emission, product quality management, and alterations required to support TDF^14–17^. Hence, pyrolysis of discarded tires is a viable alternative technology for recovering both energy and valuable compounds from the products^3,7^.

Depending on the process circumstances, pyrolysis is a thermochemical process that aims to produce various gaseous, liquid, and solid energy carriers. Pyrolysis is a technique that can be used to valorize waste tires by converting them into useful products. Pyrolysis of waste tires is increasing reputation as alternative procedure of waste tire recovering^18–20^. Pyrolysis is an inert heat activity that converts organic materials into low-molecular weight molecules^14,21^. Gas (pyrolysis gas, C1–C5), a liquid phase (oil, C6–C16), solid compounds such as metals and char or (C20–C24) is produced during pyrolysis process from the organic rubber material in waste tires^18,19,22–24^. The high volatile content of waste tires results in high yields of various products including pyrolysis gas, pyrolysis char, and pyrolysis oil^16,22^. The produced gas from pyrolysis process possesses high energy in the range of − 29.9 to 42.1 MJ m^−3^. It depends on the tire brands used in the pyrolysis process—and it is mostly used as alternative fuel in the pyrolysis process^23,25–27^. The pyrolysis char comprises the tire’s inorganic material (silicates, zinc oxide, ash, steel, etc.) as well as non-volatile carbon black^14,28,29^. After activation and upgrading, the char is able to be applied as activated carbon or fuel in the tire assembly procedure^14,28,30^. The reactor-based produced volatiles are cooled in a condenser. Then after, an oil product is achieved and separated from the non-condensable gases which is important in this process^31^. The pyrolysis oil fraction is highly attractive for pyrolysis products.

The calorific value of tire-derived oil (TDO) is approximately between 40 and 44 MJ kg^−1^ and is generally utilized as an alternative fuel, either alone or in combination with diesel^29,32–34^. TDO has a broad range of boiling points (about 50 °C to over 350 °C)^29^. The formation of treasured chemicals including xylene, styrene, benzene, toluene, ethylbenzene (BTEX) and limonene can be increased with decreasing pyrolysis pressure^10^, and temperature^35–37^. In previous studies, the impact of the pyrolysis temperature and heating rate on the chemical composition of the TDO was investigated as it has a significant role in limonene production^10,36,38–41^.

It should be noted that in the software of Aspen Plus, equilibrium reaction models (kinetic free) were used to simulate the pyrolysis process as well as the gasification of polymers, coal, biomass, and tire feeds^42–44^. However, the classification of products of the pyrolysis process was not performed in the developed model and was not related to the design equations of the process. To improve the accuracy of equilibrium-based models, several methods have been suggested (e.g., using experimental results, quasi-equilibrium temperature approach, and kinetic models). Because of the difficulties in obtaining reaction parameters from experimental data, makes the model restricted to operating conditions (e.g., specific feed). Although the quasi-equilibrium temperature approach^43^ broadens the applicability of the model, specifically for gasification simulation, it makes it less accurate for pyrolysis simulation and unable to provide enough data to justify the design equations. Kinetic models, on the other hand, are supplementary precise and more detailed than equilibrium models, but they are also more computationally precise and complicated. In the open literature, there are numerous kinetic models for the pyrolysis of various feeds^45–48^. However, due to the various and intricate dynamics involved, developing such a simulation remains a big problem, hence using rate-based kinetic model would be significantly interesting. This paper describes the development of the power-law kinetic model in Aspen Plus and the validation with literature experimental data as much as possible or created exactly for the certain systems modelled in Aspen Plus^®^ for limonene production. This work also focuses on the optimization of the operating and structure parameters of the reactor and distillation columns in the product separation unit as well as investigation to find the appropriate heating rate and temperature in terms of having the highest limonene amount in waste tire pyrolysis process.

## Modeling approach

### Conceptual process flow sheet

**Figure 1:**
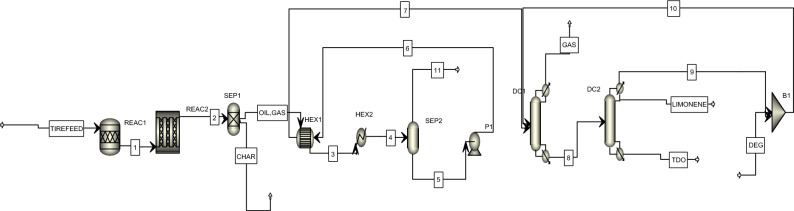
The developed simulation in software environment.

The tire pyrolysis in this research is represented in Aspen Plus simulator (Fig. 1). The conceptual process considered here is developed model from published process for pyrolytic conversion of waste tire to hydrocarbons (TDO)^35,49,50^.

The pyrolysis reaction stage was represented in the flowsheet as a mix of a stoichiometric reactor, a plug flow reactor, and distillation columns. The non-conventional solid feed elements were converted into their conventional essential element by the preset stoichiometric reactor (Reac1), which operated at 400–700 °C under 1 atm. The products exiting the stoichiometric reactor were in vapor phase except char black and metal ash. Then, the reactor, including reaction kinetic model to convert waste tire to liquid, solid and vapor was modeled. Temperatures changing from 400 to 700 °C were used to simulate pyrolysis reactions using the selected flow rate. Also, reactor dimensions which were used in the simulation were a diameter of 0.15 m and length of 1.7 m length for production of oil as well as gas products conferring to the specified kinetics in Table 1 based on Ismail et al.^35^. A separator 1 separated the non-vapor products from the vapor products.

**Table 1:** Tire pyrolysis reactions with estimated reaction constants to model tire pyrolysis process in the software of Aspen Plus^35^.

No	Name	*A*	*n*	*E* (kJ mol^−1^)
1	C + 2 H_2_ → CH_4_	4.877	0	23.01
2	2 C + 3 H_2_ → C_2_H_6_	0.52	0	23.01
3	2 C + 2 H_2_ → C_2_H_4_	2.386	0	23.01
4	3 C + 4 H_2_ → C_3_H_8_	0.277	0	23.01
5	3 C + 3 H_2_ → C_3_H_6_	0.446	0	23.01
6	4 C + 5 H_2_ → C_4_H_10_	0.122	0	23.01
7	4 C + 4 H_2_ → C_4_H_8_	0.144	0	23.01
8	4 C + 3 H_2_ → C_4_H_6_	0.981	0	23.01
9	C + O_2_ → CO_2_	0.226	0	23.01
10	C + 0.5 O_2_ → CO	0.096	0	23.01
11	H_2_ + S → H_2_S	500	0	0
12	5 C + 6 H_2_ → C_5_H_12_	0.339	0	23.01
13	5 C + 4 H_2_ → C_5_H_8_	0.066	0	23.01
14	6 C + 6 H_2_ → C_6_H_12_	0.009	0	1.59
15	6 C + 6 H_2_ → C_6_H_12_	0.009	0	1.59
16	8 C + 7 H_2_ → C_8_H_14_	0.016	0	1.59
17	8 C + 9 H_2_ → C_8_H_18_	0.023	0	1.59
18	7 C + 7 H_2_ → C_7_H_14_	0.015	0	0.006657
19	8 C + 9 H_2_ → C_8_H_18_	0.019	0	1.59
20	8 C + 9 H_2_ → C_8_H_18_	0.044	0	0.006657
21	7 C + 7 H_2_ → C_7_H_14_	0.008	0	1.59
22	7 C + 7 H_2_ → C_7_H_14_	0.045	0	0.006657
23	8 C + 8 H_2_ → C_8_H_16_	0.01	0	1.59
24	8 C + 8 H_2_ → C_8_H_16_	0.007	0	1.59
25	8 C + 7 H_2_ → C_8_H_14_	0.011	0	1.59
26	8 C + 8 H_2_ → C_8_H_16_	0.054	0	1.59
27	8 C + 8 H_2_ → C_8_H_16_	0.003	0	1.59
28	9 C + 9 H_2_ → C_9_H_18_	0.003	0	1.59
29	9 C + 9 H_2_ → C_9_H_18_	0.017	0	1.59
30	9 C + 9 H_2_ → C_9_H_18_	0.164	0	1.59
31	10 C + 8 H_2_ → D-LIM	0.035	0	1.59
32	10 C + 8 H_2_ → C_10_H_16_	0.064	0	1.59
33	10 C + 8 H_2_ → D-LIM	0.619	0	1.59
34	6 C + 3 H_2_ → C_6_H_6_	1.654	0	33.89
35	7 C + 4 H_2_ → C_7_H_8_	7.305	0	33.89
36	8 C + 5 H_2_ → C_8_H_10_	4.708	0	33.89
37	8 C + 5 H_2_ → C_8_H_10_	4.476	0	33.89
38	8 C + 4 H_2_ → C_8_H_8_	4.049	0	33.89
39	8 C + 5 H_2_ → C_8_H_10_	1.084	0	33.89
40	9 C + 6 H_2_ → C_9_H_12_	1.07	0	33.89
41	9 C + 5 H_2_ → C_9_H_10_	0.5	0	33.89
42	9 C + 6 H_2_ → C_9_H_12_	1.117	0	33.89
43	9 C + 6 H_2_ → C_9_H_12_	1.189	0	33.89
44	9 C + 6 H_2_ → C_9_H_12_	2.128	0	33.89
45	9 C + 6 H_2_ → C_9_H_12_	0.424	0	33.89
46	6 C + 3 H_2_ + O → C_6_H_6_O	0.497	0	33.89
47	9 C + 5 H_2_ → C_9_H_10_	1.532	0	33.89
48	7 C + 2.5 H_2_ + N → C_7_H_5_N	0.528	0	33.89
49	9 C + 5 H_2_ → C_9_H_10_	0.567	0	33.89
50	9 C + 6 H_2_ → C_9_H_12_	1.808	0	33.89
51	9 C + 5 H_2_ → C_9_H_10_	0.634	0	33.89
52	9 C + 5 H_2_ → C_9_H_10_	0.344	0	33.89
53	10 C + 7 H_2_ → C_10_H_14_	3.85	0	33.89
54	9 C + 6 H_2_ → C_9_H_12_	0.392	0	33.89
55	9 C + 5 H_2_ → C_9_H_10_	0.922	0	33.89
56	9 C + 4 H_2_ → C_9_H_8_	1.278	0	33.89
57	10 C + 7 H_2_ → C_10_H_14_	1.058	0	33.89
58	10 C + 7 H_2_ → C_10_H_14_	0.338	0	33.89
59	10 C + 7 H_2_ → C_10_H_14_	0.769	0	33.89
60	10 C + 7 H_2_ → C_10_H_14_	0.397	0	33.89
61	10 C + 7 H_2_ → C_10_H_14_	0.678	0	33.89
62	9 C + 5 H_2_ → C_9_H_10_	0.516	0	33.89
63	10 C + 7 H_2_ → C_10_H_14_	0.383	0	33.89
64	10 C + 7 H_2_ → C_10_H_14_	0.4	0	33.89
65	10 C + 7 H_2_ → C_10_H_14_	0.198	0	33.89
66	8 C + 5 H_2_ + 0.5 O_2_ → C_8_H_10_O	0.316	0	33.89
67	9 C + 5 H_2_ → C_9_H_10_	0.759	0	33.89
68	7 C + 3 H_2_ + O_2_ → C_7_H_6_O_2_	0.549	0	33.89
69	9 C + 5 H_2_ → C_9_H_10_	3.694	0	33.89
70	10 C + 5 H_2_ → C_10_H_10_	0.539	0	33.89
71	10 C + 6 H_2_ → C_10_H_12_	0.562	0	33.89
72	10 C + 7 H_2_ → C_10_H_14_	0.165	0	33.89
73	9 C + 5 H_2_ → C_9_H_10_	0.433	0	33.89
74	10 C + 4 H_2_ → C_10_H_8_	0.979	0	33.89
75	10 C + 7 H_2_ + 0.5 O_2_ → C_10_H_14_O	0.056	0	33.89
76	7 C + 2.5 H_2_ + 0.5 N_2_ + 2 S → C_7_H_5_NS_2_	1.2	0	33.89
77	12 C + 8 H_2_ → C_12_H_16_	47.264	− 1.089	6.3
78	12 C + 9 H_2_ → C_12_H_18_	47.815	− 1.089	6.3
79	11 C + 5 H_2_ → C_11_H_10_	125.001	− 1.089	6.3
80	11 C + 5 H_2_ → C_11_H_10_	156.807	− 1.089	6.3
81	12 C + 7 H_2_ → C_12_H_14_	9.307	− 1.089	6.3
82	12 C + 7 H_2_ → C_12_H_14_	95.891	− 1.089	6.3
83	12 C + 5 H_2_ → C_12_H_10_	142.201	− 1.089	6.3
84	12 C + 6 H_2_ → C_12_H_12_	97.289	− 1.089	6.3
85	12 C + 6 H_2_ → C_12_H_12_	37.363	− 1.089	6.3
86	12 C + 6 H_2_ → C_12_H_12_	85.169	− 1.089	0.0063
87	12 C + 6 H_2_ → C_12_H_12_	83.486	− 1.089	6.3
88	12 C + 6 H_2_ → C_12_H_12_	119.843	− 1.089	6.3
89	10 C + 4.5 H_2_ + 0.5 N_2_ → C_10_H_9_N	184.356	− 1.089	6.3
90	14 C + 14 H_2_ → C_14_ H_28_	118.294	− 1.089	6.3
91	12 C + 5 H_2_ → C_12_H_10_	36.147	− 1.089	6.3
92	15 C + 16 H_2_ → C_15_H_32_	56.974	− 1.089	6.3
93	15 C + 9 H_2_ → C_15_H_18_	88.852	− 1.089	6.3
94	15 C + 9 H_2_ → C_15_H_18_	31.429	− 1.089	6.3
95	15 C + 9 H_2_ → C_15_H_18_	29.175	− 1.089	6.3
96	13 C + 5 H_2_ → C_13_H_10_	47.77	− 1.089	6.3
97	15 C + 8 H_2_ → C_15_H_16_	60.554	− 1.089	6.3
98	15 C + 8 H_2_ → C_15_H_16_	11.521	− 1.089	6.3
99	15 C + 15 H_2_ → C_15_H_30_	17.88	− 1.089	6.3
100	16 C + 17 H_2_ → C_16_H_34_	46.822	− 1.089	6.3
101	14 C + 5 H_2_ → C_14_H_10_	34.666	− 1.089	6.3
102	14 C + 5 H_2_ → C_14_H_10_	38.059	− 1.089	6.3
103	15 C + 6 H_2_ → C_15_H_12_	36.925	− 1.089	6.3
104	15 C + 15 H_2_ + O_2_ → C_15_H_30_O_2_	64.017	− 1.089	6.3
105	15 C + 6 H_2_ → C_15_H_12_	41.028	− 1.089	6.3
106	15 C + 6 H_2_ → C_15_H_12_	46.908	− 1.089	6.3
107	15 C + 6 H_2_ → C_15_H_12_	82.056	− 1.089	6.3
108	19 C + 20 H_2_ → C_19_H_40_	12.247	− 1.089	6.3
109	19 C + 8 H_2_ → C_19_H_16_	22.599	− 1.089	6.3
110	19 C + 19 H_2_ → C_19_H_38_	51.627	− 1.089	6.3
111	20 C + 21 H_2_ → C_20_H_42_	13.594	− 1.089	6.3
112	21 C + 22 H_2_ → C_21_H_44_	15.524	− 1.089	6.3
113	22 C + 23 H_2_ → C_22_H_46_	12.028	− 1.089	6.3
114	23 C + 24 H_2_ → C_23_H_48_	15.641	− 1.089	6.3
115	24 C + 25 H_2_ → C_24_H_50_	3.029	− 1.089	6.3
116	11 C + 12 H_2_ → C_11_H_24_	35.684	− 1.089	6.3

In a heat exchanger 1 and cooler 1, cooling water was used for the cooling of the vapor product from 400–700 °C to 35 °C, and it was chilled to lower its boiling temperature. A separator 2 separated the stream into a liquid comprising oil products as well as a vapor phase carrying non-condensable gas products. There was no solid material predicted in the oil feed to separator 2 (knocked out drum) that may cause blocking of the trays or loading substance in the separation columns^51^. The pyrolysis section's oil supply stream is pushed to 200 kPa. Before being fed into the first distillation column, the oil is compressed, and the compounds lighter than the limonene cut compound are released as vapor. The bottoms stream is then delivered to the second distillation column, where the components weightier than the limonene cut are released as bottoms products (heavy TDO), leaving just the limonene-rich cut as the liquid distillate product. Diethylene glycol was added to the second distillation column to eliminate the majority of the impurities, yielding a limonene distillate with (minimum) 95 weight percent limonene purity.

In the research of Ngwetjana^52^, a selection of candidate entrainers was identified. The investigated entrainers by Ngwetjana^52^ included diethylene glycol (DEG), triethylene glycol (TEG), n,n-dimethylformamide (DMF), n-methyl-2-pyrrolidone (MP), quinoline, 4-formylmorpholine (4-FM) and tetratethylene glycol dimethyl ether (TEDE). RCM technology was employed to determine entrainer feasibility by showing alteration of the relative volatility of the dl-limonene and p-cymene mixture and ability in their separation. DEG was introduced as a probable entrainer as it resulted in the creation of heterogeneous azeotropes facilitating the separation of d-limonene and p-cymene. TEG eventuated in the formation of a region for liquid–liquid de-mixing, allowing the crossing of the distillation boundary. TEG was also considered as a an efficient possible entrainer. The choice between DEG and TEG was based on process economics. 4-FM could be known as a probable entrainer as it resulted in the formation of heterogeneous azeotropes, facilitating the separation of d-limonene and p-cymene but had a binodal curve (liquid–liquid solubility) smaller than that observed in TEG and DEG. DMF, Quinoline and MP were not regarded as feasible entrainer. TEDE was not formed any azeotrope with any of the components.

Among the investigated entrainers, diethylene glycol was selected as it has a high boiling entrainer and introduces a heterogeneous azeotrope when employed as an entrainer along with economic matter.

### Feed of the reaction model and reaction kinetics

Decompositions of big hydrocarbon chains into lesser particles are the reactions that occur.

The feed in Aspen Plus is determined by its essential constituents rather than its chemical structure.

The following kinetic model from Ismail et al.^35^ and Olazar et al.^25^ were used in this paper

1 $$\frac{{dX_{n} }}{dt} = \left( {k_{g} + k_{l} + k_{a} + k_{i} } \right)\left( {1 - X_{n} } \right)$$

2 $$\frac{{dX_{g} }}{dt} = \left( {k_{g} } \right)\left( {1 - X_{n} } \right)$$

3 $$\frac{{dX_{l} }}{dt} = \left( {k_{l} } \right)\left( {1 - X_{n} } \right)$$

4 $$\frac{{dX_{a} }}{dt} = \left( {k_{a} } \right)\left( {1 - X_{n} } \right) + k_{ia} X_{i}$$

5 $$\frac{{dX_{t} }}{dt} = \left( {k_{it} X_{i} } \right)$$

6 $$\frac{{dX_{c} }}{dt} = \left( {k_{ic} X_{i} } \right)$$

7 $$\frac{{dX_{i} }}{dt} = k_{i} \left( {1 - X_{n} } \right) - k_{ia} X_{i} - k_{it} X_{i} - k_{ic} X_{i}$$

where X_n_ = Overall mass conversion (kg converted/kg initial); X_g_; X_l_; X_a_; X_t_; X_c_; X_i_ = Mass fraction gas yield of gas, oil, aromatics, tar, char, and intermediates, respectively; k_g_; k_l_; k_a_; k_i_; k_ia_; k_it_; k_ic_ = Rate constants for tire-gas, tire-liquid, tire-aromatic, tire-intermediate, intermediate-aromatic, intermediate-tar, and intermediate-char kinetic respectively.

Then, intermediate component terms by assuming pseudo-steady state condition were eliminated $\left( {\frac{{dX_{i} }}{dt} = 0} \right)$, next, X_i_ in terms of X_n_ were taken and substituted in the initial kinetic models (Eqs. 1–7). After that X’_n_ = 1-X_n_ to find the mass percentage time remaining and lump all kinetic rate constants to change equations to the first-order kinetic model. Finally, Arrhenius parameters were estimated from these first-order equations for rate constants and the following rate equations were obtained.

8 $$C1 - C4 = 0.0283 e^{{ - \frac{23010}{{RT}}}} X_{n}^{\prime } \left( {{\text{gas}}} \right)$$

9 $$C5 - C10\left( {non{\text{-}}aromatics} \right) = 0.014 e^{{ - \frac{1590}{{RT}}}} X_{n}^{\prime } \left( {{\text{oil}}} \right)$$

10 $$C5 - C10\left( {aromatics} \right) = 0.5895 e^{{ - \frac{32890}{{RT}}}} X_{n}^{\prime } \left( {{\text{oil}}} \right)$$

11 $$C11 + \left( {aromatics\;\& \;non{\text{-}}aromatics} \right) = 37.61 T^{ - 1.089} e^{{ - \frac{6300}{{RT}}}} X_{n}^{\prime } \left( {{\text{oil}}} \right)$$

It is necessary to define mass conversion “X” in terms of its constituent elements. Since hydrogen makes up 7 weight percentage of the feed and is the limiting reactant, it is appropriate to substitute the mass conversion of hydrogen (H_2_) for the mass conversion of tire feed (X_n_) in Eqs. 8–11. In order to substitute ${\text{X}}_{{\text{n}}}^{\prime }$ with X_H2_, the original rate equation in terms of ${\text{X}}_{{\text{n}}}^{\prime }$ is divided by 0.07 instead.

12 $$C1 - C4 = 0.40428 e^{{ - \frac{23010}{{RT}}}} X_{H2} \left( {{\text{gas}}} \right)$$

13 $$C5 - C10\left( {non{\text{-}}aromatics} \right) = 0.2 e^{{ - \frac{1590}{{RT}}}} X_{H2} \left( {{\text{oil}}} \right)$$

14 $$C5 - C10\left( {aromatics} \right) = 8.4214 e^{{ - \frac{32890}{{RT}}}} X_{H2} \left( {{\text{oil}}} \right)$$

15 $$C11 + \left( {aromatics\;\& \;non{\text{-}}aromatics} \right) = 537.28 T^{ - 1.089} e^{{ - \frac{6300}{{RT}}}} X_{H2} \left( {{\text{oil}}} \right)$$

The formulas in Eqs. 12–15 estimate the rate expression of various products (116 compounds), as given in Table 1, and they take the Arrhenius form, for reaction i shown in Eq. 16

16 $$Ki = Ai T^{n} e^{{ - \frac{Ei}{{RT}}}} \;{\text{rate}} = {\text{K}}_{{\text{i}}} .\;{\text{individual}}\;{\text{component}}\;{\text{fraction}}$$

where the constant of A, E (kJ/mol) as activation energy, and n of the Arrhenius equation are all computed for temperatures between 400 and 700 °C. Tire was characterized with the following Proxanal and Ultanal attributes (Tables 2a, b) to the product gas, oil, char, and metal^35,51,53,54^.

**Table 2:** (**a**) Ultanal and Proxanal assessment of the tire, (**b**) Proxanal and Ultanal assessment of the char.

Element	wt%	Compound	wt%
(a)			
C	75	Ash	13.5
H	7	Moisture	1.5
N	0.3	Fixed carbon	30
O	2.7	Volatile materia	55
S	1.5	–	–
(b)			
C	82.76	Ash	12.21
H	0.55	Moisture	0
N	0.44	Fixed carbon	87.78
S	4.03	Volatile materia	0
Ash	12.21	–	–

### Thermodynamic models

The remainder of Fig. 1’s process simulation was created using native Aspen Plus unit operation blocks^35,49,50^. To determine the physical characteristics for all the prevalent components in the current investigation, the Peng-Robinson with a Boston-Mathias alpha function (PR-BM) property technique was selected. HCOALGEN and DCOALIGT property models were applied for the enthalpy/density calculation of tire and char^36,52^. Thermodynamic properties of components were estimated applying the non-random two-liquid (NRTL). in the current study's solvent recovery portion and UNIFAC property model utilized the missing values of NRTL^55,56^.

For non-ideal liquid mixes, activity coefficient property models are advised, and solvent recovery techniques are advocated in the literature^57^. Activity coefficient models are precise for phase equilibrium computations when binary contact factors are given. In the absence of vapor–liquid equilibrium (VLE) data, the UNIFAC predictive model can be used to assess the needed constraints and create the binary parameters^51^.

### Heating rate and pressure

Temperature, pressure, and heating rate are the primary aspects that determine waste tire pyrolysis^58,59^. The reaction rate and heating profile is influenced by the heating rate in the elements, therefore it is an important variable in pyrolysis^4,60^. The yield of aliphatic compounds boosted as the heating rate was improved, but the yield of aromatic products decreased^61^. When the rate of reaction was raised, higher heating rates were beneficial for the creation of limonene; nevertheless, quicker elimination of primary volatiles was necessary to reduce the happening of secondary reactions that reduce limonene^10,37,39,51^. Consequently, determining the optimal heating rate was important in the pyrolysis process. The maximum efficiency of oil was found at a heating rate of 10 °C/min among 5, 10, 15, and 20 °C/min, as an example^38^. On the other hand, Williams and Brindle^37^ investigated the cause of adjusting the heating rate from 1 to 80 °C/min and discovered that the highest oil heating rate was achieved at 15 °C/min^54^. Several investigations in the literature have used a pyrolysis pressure = 100 kPa as the optimal working pressure^33,36,62–65^. Pyrolysis in vacuum lowered volatile residence duration by enhancing diffusion of volatiles to the outside of the tire element due to the produced positive pressure gradient^18,65^. A rise in residence time generated a growth in gas yield at the price of oil efficiency because of longer residence times promoting the happening of secondary reactions and breaking of the oil product into gas^58,66,67^. Increased volatiles residence time may result in a decline in char yield because of high contact times of the char product with volatile compounds, that could result in secondary reactions such the Boudouard reaction^58,66^.

## Results and discussion

### Influence of DEG amount

**Figure 2:**
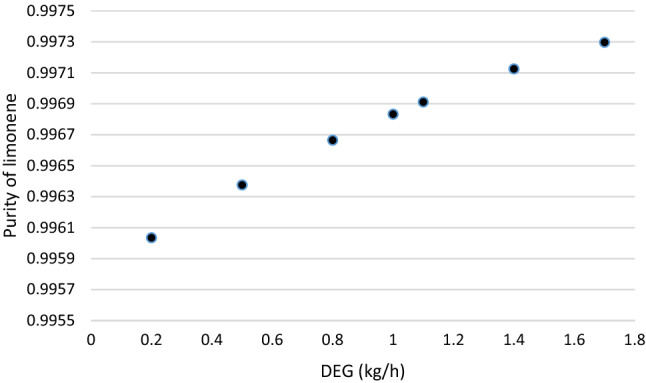
Effect of DEG amount on limonene purity.

The remnant limonene in extract stream of second distillation column, where DEG is created to eliminate remnant impurities is recycled to first distillation column. Limonene was increased with increasing DEG, Fig. 2. In the current work, the second distillation process used a solvent to investigate the recovery of limonene from TDO as high as possible^68^. The process of increasing limonene recovery from TDO using DEG as the solvent is used in this study. Increasing amount of DEG has better effect on limonene purity (Fig. 2).

### Effect of stage

The difficulties of splitting p-cymene and limonene by conventional distillation was demonstrated in the experimental work^10^. As a result, improved distillation methods are needed to split these two components, which is why extractive distillation was used in this study to recover limonene from the limonene-rich stream. In this investigation a straightforward process design was done rather than a big, complex system with numerous process steps to create, recover, and purify a variety of products.

**Figure 3:**
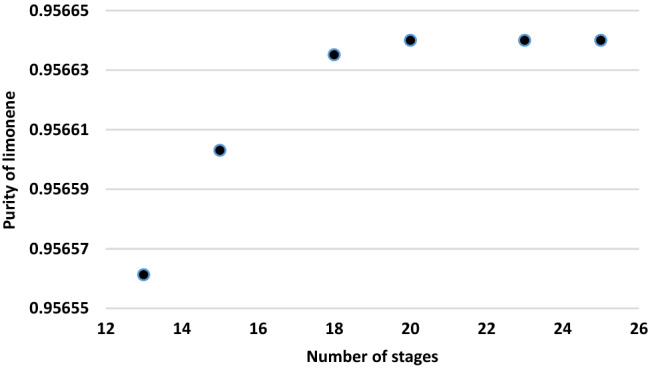
Effect of number of stages of first distillation column on limonene purity.

There is no solid material in the oil feed to the distillation column that might cause blocking of the trays or filling substances in the distillation column. A RADFRAC distillation column model was also used to model the first distillation column. The supreme constraints are a reflux ratio of 11, a distillate/feed = 0.2, and a feed location at stage 9 based on the findings of sensitivity analysis at number of stages of 20. The heavy TDO was almost unchanged with changing number of stages in column 1. The changes number of stages from 13 to 20 increased limonene purity slowly and more than 20 stages was almost constant (Fig. 3). The increase in limonene recovery was attributed to the inclusion of more stages in the stripping part of the column when feed stage is fixed, allowing for additional interaction with the hot vapors and thus increased limonene stripping.

**Figure 4:**
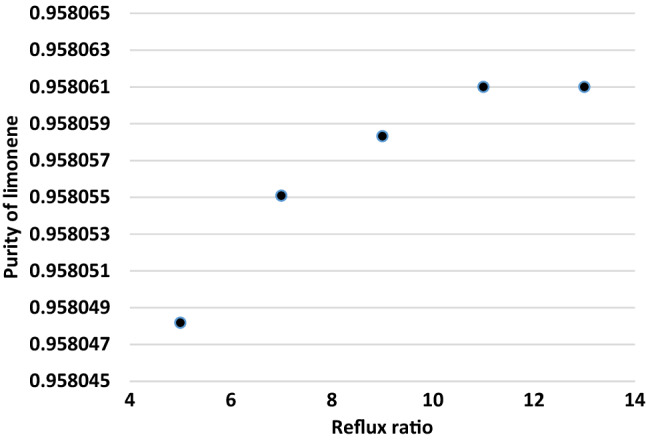
Impact of reflux ratio on the purity value of limonene.

**Figure 5:**
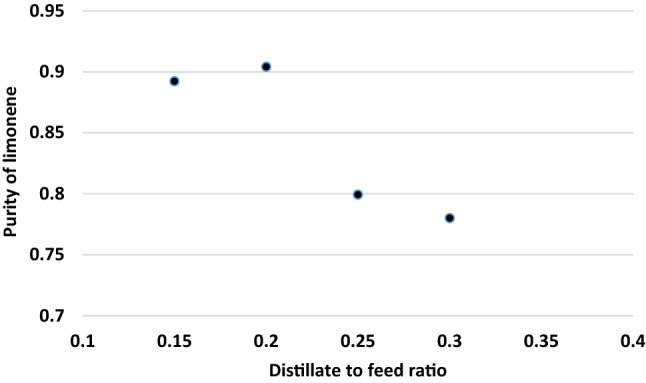
Impact of distillate-to-feed ratio on the purity value of limonene.

Increasing the reflux ratio caused to decrease the recovery rate of limonene. It was shown in Fig. 4. The decrease in limonene vaporization assigned to the reboiler's lowering energy input to fulfill the reducing refluxing needs. As a result, the limonene was stripped less, resulting in a high limonene recovery in the bottoms product. Furthermore, Fig. 5 illustrates the influence of distillate-to-feed ratio on limonene purity. It was found that there is a bit increase in the limonene purity when distillate-to- feed ratio enhanced from 0.15 to 0.2. However, it was decreased from about 0.9 to 0.78 with the enhancement of distillate to feed ratio from 0.20 to 0.30.

The final RADFRAC column parameters for the first distillation column are illustrated in Table 3a. Atmospheric pressure is used in the second distillation column, and the condenser pressure is set at 100 kPa. The first column should ideally be a packed column. Packed columns are ideal for insignificant diameters, temperature-delicate items, and challenging separations requiring multiple stages^69,70^. Structured packing is advantageous for these activities because it can provide a height equal to theoretical plate (HETP) of less than 0.5 m and a minimal pressure drop (less than 100 Pa/m)^71^. As a result, based on these HETP, a stage pressure drop of 50 Pa was established.

**Table 3:** (**a**) Final RADFRAC column parameters for first distillation column, (**b**) Final operating parameters for the second distillation column.

Parameter	Value
(a)	
Number of stages	20
Reflux ratio	11-mass
Distillate/waste tire	0.2-mol
Feed stage	9
Feed temperature	170 °C
(b)	
Number of stages	18
Reflux ratio	3-mass
Boil up ratio	4-mass
Feed stage	9
Feed temperature	170 °C

Despite the significant reduction in pressure drop, working at atmospheric pressure results in a reboiler temperature of roughly 210 °C. It should be remarked that at these temperatures, thermal breakdown of certain components may occur, resulting in packing material fouling. Because the effective cross-sectional area available to vapor flow affects the capacity of a packed column, this would diminish separation capacity^69,70,72,73^. The impacts of hold-up must be evaluated in such circumstances. When compared to plate columns, liquid hold-up is usually much lower for packed columns^71^. Final operating parameters for the second column were shown in Table 3b.

**Figure 6:**
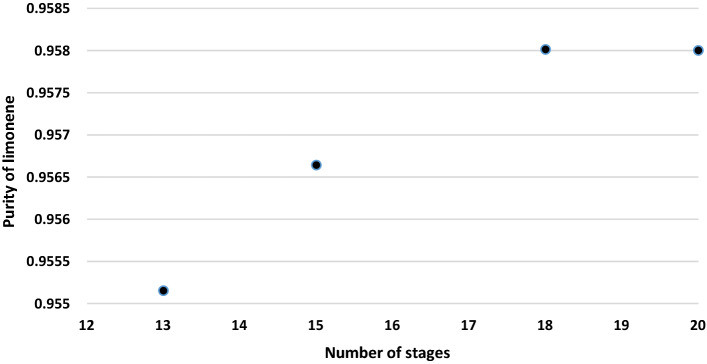
Influence of number of stages of second column on limonene purity.

As shown in Fig. 6, the recovery of limonene increased with increasing stages number and 18 stages as an optimum stage was selected to be able to divide limonene from other TDO.

**Figure 7:**
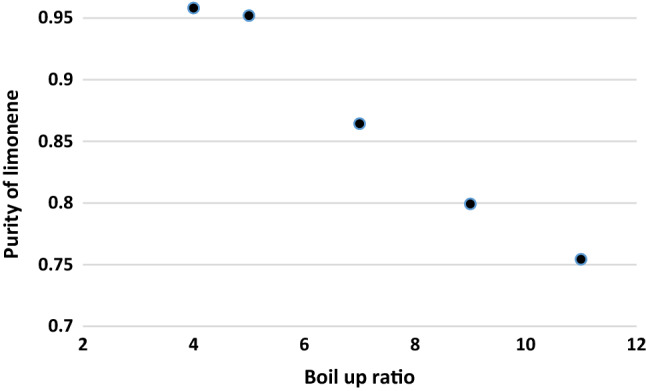
Impact of biol-up ratio on limonene purity.

Influence of biol-up ratio on limonene purity was demonstrated in Fig. 7. Limonene recovery decreased with a rise in boil up ratio for reflux ratios below 10 as more bottom’s product is vaporized and reverted as boil up. Number of stages were not so effective on limonene purity, Fig. 6. For reflux ratios below 10, limonene recovery diminishes as the boil-up ratio rises, since more bottom product is vaporized and recovered as boil-up, Fig. 7. There is no difference in recovery at a reflux ratio of ten because the greater reflux ratio counteracts the effects of risen boil-up^44^.

### Effect of temperature on TDO, limonene and gases

The most typical temperatures for waste tire pyrolysis are 425 °C to 600 °C, with 500 °C being the most prevalent in the assortment^22,26,29,36^. By enhancing the pyrolysis temperature from 450 to 475 °C, Cunliffe and Williams^21^ noticed that the oil output rose and peaked at 475 °C^63^. Cunliffe and Williams^21^ found that oil yield declined as temperature increased from 475 to 600 °C, with a comparable rise in gas yield^63^. Secondary reactions involving the breakdown of higher molecular types into gaseous products were also blamed. The best temperature for oil yield was 450 °C, which was chosen from 400, 450, and 500°C^38^. The ultimate pyrolysis temperature was enhanced from 500 to 700 °C, and the aliphatic fraction concentration in the pyrolysis oil reduced from 15.1 to 6.1 wt%, while the aromatic fraction concentration rose from 65.3 to 79.3 wt%^36^. When the temperature rises, the production of limonene drops, but the yield of aromatic chemicals like BTX rises noticeably. At temperatures exceeding 500 °C, limonene decomposes into aromatics including toluene, trimethylbenzene, xylene, m-cymene, benzene, and indane^37,39^.

By repeating the simulation at various temperatures and monitoring the quantities of products generated, the effect of temperature was investigated. In this research, as it is shown in Table 4, the highest limonene and TDO was obtained at 500 °C and gas amount was increased by increasing temperature while char was decreasing by increasing temperature. The similar results were obtained by other researches^10,22,63,74^.

**Table 4:** Results of this simulation at different temperatures for tire pyrolysis products.

Temperature (°C)	Char (kg h^−1^)	Gas (kg h^−1^)	Limonene (kg h^−1^)	TDO (kg h^−1^)
400	24.36	14.20	38.40	39.95
450	24.20	14.31	39.04	38.76
500	24.00	14.45	39.51	37.78
600	23.59	14.77	39.18	37.39
700	23.14	14.98	39.13	37.17

The result differences in different temperatures in this simulation were similar to the experimental results of Choi et al.^36^. Table 5 compares the simulated and experimental results at the heating rate and temperature of 10 C min^−1^ and 450 °C, respectively. As can be seen in this table, the liquid percentage significantly increased. Higher liquid percentage was obtained in this simulation compared with the experiment of Uyumaz et al. because of the selected separation methods^38^. It will be recommended to do the experiment of this modelling in future.

**Table 5:** Simulated and experimental results at 450 °C and heating rate of 10 C min^−1^.

At temperature of 450 (°C)	Char (wt%)	Gas (wt%)	Liquid (limonene + TDO) (wt%)
Experimental^38^	8	36	56
Simulation of this study	12	21	67

For the main oil products, gasoline and diesel, an energy analysis was carried out at a wide range of temperature (300–700 °C) to ascertain the process efficiency. Both gasoline and diesel contain hydrocarbons in the C_4_–C_10_ range as well as those in the C_11_–C_21_ range respectively. The liquid and char products are reduced as the temperature rises, but the aromatic and gas products are increased. Owing to the fact that diesel is made up of large hydrocarbon chains, the decomposition of these chains occurs more frequently as the temperature rises, as shown in Table 4. This correlates to the reduction in the combustion power produced by diesel. While the composition of gasoline with shorter hydrocarbon chains grows as the temperature rises, increasing the amount of combustion power. The net power generated improves with temperature and greater values of energy is accessible for this reforming. It is primarily caused by the cracking of large chains into smaller ones and the reformation of the smaller chains (liquid) to their corresponding aromatic structures.

## Conclusion

A model-based investigation of waste tire pyrolysis is presented in this paper. The pyrolysis process products were predicted using a flowsheet simulation under various operational circumstances such as reactor temperature, number of stages in the distillation column, and so on. It was validated against experimental data^36,38^. The simulation model was able to accurately forecast the hydrocarbon product mass fractions. The bigger hydrocarbon chains were broken into smaller ones at the optimal temperature and heating rate, as evidenced by reduced mass fractions of C10–C15 and greater mass fractions of C7–C9. Furthermore, more aromatics would be created, with less tar and non-aromatics. In addition, operating at low temperatures was found to be the most energy effective from a net energy aspect, with the largest quantity of diesel produced and the least amount of gasoline produced. Then, using the construction of two distillation columns to separate gas, limonene, and TDO from each other, it was attempted to attain high purity of limonene. The simulation model given in this work presents itself as a tool that will help pyrolysis plant operators to adapt to market changes in a cost-effective manner by identifying the most cost-effective operating temperatures.

## Data Availability

All data generated or analyzed during this study are included in this article.

## References

[1] Pan Y, Sima J, Wang X, Zhou Y, Huang Q (2021). BTEX recovery from waste rubbers by catalytic pyrolysis over Zn loaded tire derived char. Waste Manag..

[2] Putman, B. J. & Amirkhanian, S. N. in *Proceedings of the Asphalt Rubber 2006 Conference* 655–677.

[3] Murillo R (2006). The application of thermal processes to valorise waste tyre. Fuel Process. Technol..

[4] Martínez JD (2013). Waste tyre pyrolysis—A review. Renew. Sustain. Energy Rev..

[5] De D, De D (2011). Processing and material characteristics of a reclaimed ground rubber tire reinforced styrene butadiene rubber. Mater. Sci. Appl..

[6] Ucar S, Karagoz S, Ozkan AR, Yanik J (2005). Evaluation of two different scrap tires as hydrocarbon source by pyrolysis. Fuel.

[7] Seidelt S, Müller-Hagedorn M, Bockhorn H (2006). Description of tire pyrolysis by thermal degradation behaviour of main components. J. Anal. Appl. Pyrol..

[8] Karger-Kocsis J, Mészáros L, Bárány T (2013). Ground tyre rubber (GTR) in thermoplastics, thermosets, and rubbers. J. Mater. Sci..

[9] Smith A, Ade H, Koch C, Spontak R (2001). Cryogenic mechanical alloying as an alternative strategy for the recycling of tires. Polymer.

[10] Pakdel H, Pantea DM, Roy C (2001). Production of dl-limonene by vacuum pyrolysis of used tires. J. Anal. Appl. Pyrol..

[11] Taleghani AS (2021). Mesoporous silica nanoparticles as a versatile nanocarrier for cancer treatment: A review. J. Mol. Liq..

[12] Rofiqul IM, Haniu H, Rafiqul ABM (2007). Limonene-rich liquids from pyrolysis of heavy automotive tire wastes. J. Environ. Eng..

[13] Pan Y (2022). Catalytic co-pyrolysis of rubber waste and polyacrylonitrile for producing BTEX enriched oil via heterogeneous Diels-Alder reaction. Fuel.

[14] Amari T, Themelis NJ, Wernick IK (1999). Resource recovery from used rubber tires. Resour. Policy.

[15] Giugliano M, Cernuschi S, Ghezzi U, Grosso M (1999). Experimental evaluation of waste tires utilization in cement kilns. J. Air Waste Manag. Assoc..

[16] Conesa JA, Gálvez A, Mateos F, Martín-Gullón I, Font R (2008). Organic and inorganic pollutants from cement kiln stack feeding alternative fuels. J. Hazard. Mater..

[17] Ghadiri M, Hemmati A, Nakhjiri AT, Shirazian S (2020). Modelling tyramine extraction from wastewater using a non-dispersive solvent extraction process. Environ. Sci. Pollut. Res..

[18] López G, Olazar M, Aguado R, Bilbao J (2010). Continuous pyrolysis of waste tyres in a conical spouted bed reactor. Fuel.

[19] Islam M, Joardder M, Hasan S, Takai K, Haniu H (2011). Feasibility study for thermal treatment of solid tire wastes in Bangladesh by using pyrolysis technology. Waste Manag..

[20] Cao Y, Nakhjiri AT, Ghadiri M (2022). Membrane desalination for water treatment: Recent developments, techno-economic evaluation and innovative approaches toward water sustainability. Eur. Phys. J. Plus.

[21] Cunliffe A, Williams P (1998). Properties of chars and activated carbons derived from the pyrolysis of used tyres. Environ. Technol..

[22] Wang W-C, Bai C-J, Lin C-T, Prakash S (2016). Alternative fuel produced from thermal pyrolysis of waste tires and its use in a DI diesel engine. Appl. Therm. Eng..

[23] Kyari M, Cunliffe A, Williams PT (2005). Characterization of oils, gases, and char in relation to the pyrolysis of different brands of scrap automotive tires. Energy Fuels.

[24] Pishnamazi M (2020). Computational investigation on the effect of [Bmim][BF4] ionic liquid addition to MEA alkanolamine absorbent for enhancing CO_2_ mass transfer inside membranes. J. Mol. Liq..

[25] Olazar M (2008). Catalyst effect on the composition of tire pyrolysis products. Energy Fuels.

[26] Williams PT, Besler S (1995). Pyrolysis-thermogravimetric analysis of tyres and tyre components. Fuel.

[27] Nakhjiri AT, Roudsari MH (2016). Modeling and simulation of natural convection heat transfer process in porous and non-porous media. Appl. Res. J..

[28] Gao N (2022). Tire pyrolysis char: Processes, properties, upgrading and applications. Prog. Energy Combust. Sci..

[29] Li S-Q, Yao Q, Chi Y, Yan J-H, Cen K-F (2004). Pilot-scale pyrolysis of scrap tires in a continuous rotary kiln reactor. Ind. Eng. Chem. Res..

[30] Wójtowicz MA, Serio MA (1996). Pyrolysis of scrap tires: Can it be profitable?. CHEMTECH-WASHINGTON DC.

[31] Farzad S, Mandegari M, Görgens JF (2021). A novel approach for valorization of waste tires into chemical and fuel (limonene and diesel) through pyrolysis: Process development and techno economic analysis. Fuel Process. Technol..

[32] Aydın H, İlkılıç C (2015). Analysis of combustion, performance and emission characteristics of a diesel engine using low sulfur tire fuel. Fuel.

[33] Frigo S, Seggiani M, Puccini M, Vitolo S (2014). Liquid fuel production from waste tyre pyrolysis and its utilisation in a Diesel engine. Fuel.

[34] Cao Y (2021). Recent advancements in molecular separation of gases using microporous membrane systems: A comprehensive review on the applied liquid absorbents. J. Mol. Liq..

[35] Ismail HY, Abbas A, Azizi F, Zeaiter J (2017). Pyrolysis of waste tires: A modeling and parameter estimation study using Aspen Plus^®^. Waste Manag..

[36] Choi G-G, Jung S-H, Oh S-J, Kim J-S (2014). Total utilization of waste tire rubber through pyrolysis to obtain oils and CO_2_ activation of pyrolysis char. Fuel Process. Technol..

[37] Williams PT, Brindle AJ (2003). Temperature selective condensation of tyre pyrolysis oils to maximise the recovery of single ring aromatic compounds☆. Fuel.

[38] Uyumaz A (2019). Production of waste tyre oil and experimental investigation on combustion, engine performance and exhaust emissions. J. Energy Inst..

[39] Danon B, Van Der Gryp P, Schwarz C, Görgens J (2015). A review of dipentene (dl-limonene) production from waste tire pyrolysis. J. Anal. Appl. Pyrol..

[40] Čepić Z (2021). Experimental analysis of temperature influence on waste tire pyrolysis. Energies.

[41] Babanezhad M (2020). High-performance hybrid modeling chemical reactors using differential evolution based fuzzy inference system. Sci. Rep..

[42] Zhu L, Zhang L, Fan J, Jiang P, Li L (2016). MSW to synthetic natural gas: System modeling and thermodynamics assessment. Waste Manag..

[43] Visconti, A., Miccio, M. & Juchelkova, D. Equilibrium-based simulation of lignocellulosic biomass pyrolysis via Aspen Plus. In *Recent Advances in Applied Mathematics, Modelling and Simulation* 22–24 (2014).

[44] Cao Y (2021). Mathematical modeling and numerical simulation of CO2 capture using MDEA-based nanofluids in nanostructure membranes. Process Saf. Environ. Prot..

[45] Sharma RK, Yang J, Zondlo JW, Dadyburjor DB (1998). Effect of process conditions on co-liquefaction kinetics of waste tire and coal. Catal. Today.

[46] Mazloom G, Farhadi F, Khorasheh F (2009). Kinetic modeling of pyrolysis of scrap tires. J. Anal. Appl. Pyrol..

[47] Olazar M (2008). Kinetic modelling of tyre pyrolysis in a conical spouted bed reactor. J. Anal. Appl. Pyrol..

[48] Cao Y, Nakhjiri AT, Sarkar SM, Ghadiri M (2022). Time-dependent numerical investigation of 3-hydroxypropionic acid extraction using a microporous membrane contactor. Eur. Phys. J. Plus.

[49] Taylor R, Ray R, Chapman C (2013). Advanced thermal treatment of auto shredder residue and refuse derived fuel. Fuel.

[50] Altayeb, R. K. *Liquid Fuel Production from Pyrolysis of Waste Tires: Process Simulation, Exergetic Analysis, and Life Cycle Assessment* (2015).

[51] Mulaudzi L (2017). Process Modelling and Economic Evaluation of Waste Tyres to Limonene via Pyrolysis.

[52] Ngwetjana MM (2017). Fractionation of Tyre Derived Oil.

[53] Quek A, Balasubramanian R (2012). Mathematical modeling of rubber tire pyrolysis. J. Anal. Appl. Pyrol..

[54] Laresgoiti M (2004). Characterization of the liquid products obtained in tyre pyrolysis. J. Anal. Appl. Pyrol..

[55] Brondani LB, Flôres GB, Soares RDP (2015). Modeling and simulation of a benzene recovery process by extractive distillation. Braz. J. Chem. Eng..

[56] Ko MS, Na S, Cho J, Kim H (2002). Simulation of the aromatic recovery process by extractive distillation. Korean J. Chem. Eng..

[57] Chen C-C, Mathias PM (2002). Applied thermodynamics for process modeling. Am. Inst. Chem. Eng. AIChE J..

[58] González JF, Encinar JM, Canito JL, Rodriguez JJ (2001). Pyrolysis of automobile tyre waste. Influence of operating variables and kinetics study. J. Anal. Appl. Pyrol..

[59] Hopa, D. Y., Yılmaz, A. & Aksoy Bahtlı, T. Recovery of waste tyres by pyrolysis in a fixed bed reactor for liquid fuel production: Effects of pyrolysis conditions on oil yield. (2017).

[60] Martínez JD, Murillo R, García T, Veses A (2013). Demonstration of the waste tire pyrolysis process on pilot scale in a continuous auger reactor. J. Hazard. Mater..

[61] Leung D, Wang C (2003). Fluidized-bed gasification of waste tire powders. Fuel Process. Technol..

[62] Conesa JA, Martín-Gullón I, Font R, Jauhiainen J (2004). Complete study of the pyrolysis and gasification of scrap tires in a pilot plant reactor. Environ. Sci. Technol..

[63] Ozcan H, Ongen A, Pangaliyev Y (2016). An experimental study of recoverable products from waste tire pyrolysis. Global NEST J..

[64] Benallal B, Roy C, Pakdel H, Chabot S, Poirier M (1995). Characterization of pyrolytic light naphtha from vacuum pyrolysis of used tyres comparison with petroleum naphtha. Fuel.

[65] Zhang X, Wang T, Ma L, Chang J (2008). Vacuum pyrolysis of waste tires with basic additives. Waste Manag..

[66] Islam MR, Tushar M, Haniu H (2008). Production of liquid fuels and chemicals from pyrolysis of Bangladeshi bicycle/rickshaw tire wastes. J. Anal. Appl. Pyrol..

[67] Babanezhad M (2020). Liquid temperature prediction in bubbly flow using ant colony optimization algorithm in the fuzzy inference system as a trainer. Sci. Rep..

[68] Ngwetjana, M. M. Fractionation of tyre derived oil (TDO). MEng thesis, Department of Process Engineering, Stellenbosch University (2017).

[69] Mackowiak J (2010). Fluid Dynamics of Packed Columns.

[70] Berger TA (1995). Packed Column SFC.

[71] Towler G, Sinnott R (2021). Chemical Engineering Design: Principles, Practice and Economics of Plant and Process Design.

[72] Babanezhad M, Taghvaie Nakhjiri A, Rezakazemi M, Shirazian S (2020). Developing intelligent algorithm as a machine learning overview over the big data generated by Euler–Euler method to simulate bubble column reactor hydrodynamics. ACS Omega.

[73] Pishnamazi M, Taghvaie Nakhjiri A, Rezakazemi M, Marjani A, Shirazian S (2020). Mechanistic modeling and numerical simulation of axial flow catalytic reactor for naphtha reforming unit. PLoS ONE.

[74] Niksiar A, Sohrabi M, Rahimi A (2013). A correction on a published kinetic model for tyre pyrolysis in a conical spouted bed reactor. J. Anal. Appl. Pyrol..

